# Determination of formation constants and structural characterization of cyclodextrin inclusion complexes with two phenolic isomers: carvacrol and thymol

**DOI:** 10.3762/bjoc.12.5

**Published:** 2016-01-08

**Authors:** Miriana Kfoury, David Landy, Steven Ruellan, Lizette Auezova, Hélène Greige-Gerges, Sophie Fourmentin

**Affiliations:** 1 Bioactive Molecules Research Group, Doctoral School of Science and Technology, Department of Chemistry and Biochemistry, Faculty of Sciences, section II, Lebanese University, Lebanon; 2 Unité de Chimie Environnementale et Interactions sur le Vivant (UCEIV, EA 4492), ULCO, F-59140 Dunkerque, France

**Keywords:** cyclodextrins, DOSY-NMR, formation constant, molecular modeling, solubility

## Abstract

Carvacrol and thymol have been widely studied for their ability to control food spoilage and to extend shelf-life of food products due to their antimicrobial and antioxidant activities. However, they suffer from poor aqueous solubility and pronounced flavoring ability that limit their application in food systems. These drawbacks could be surpassed by encapsulation in cyclodextrins (CDs). Applications of their inclusion complexes with CDs were reported without investigating the inclusion phenomenon in deep. In this study, inclusion complexes were characterized in terms of formation constants (*K*_f_), complexation efficiency (CE), CD:guest molar ratio and increase in bulk formulation by using an UV–visible competitive method, phase solubility studies as well as ^1^H and DOSY ^1^H NMR titration experiments. For the first time, a new algorithmic treatment that combines the chemical shifts and diffusion coefficients variations for all guest protons was applied to calculate *K*_f_. The position of the hydroxy group in carvacrol and thymol did not affect the stoichiometry of the inclusion complexes but led to a different binding stability with CDs. 2D ROESY NMR experiments were also performed to prove the encapsulation and illustrate the stable 3D conformation of the inclusion complexes. The structural investigation was accomplished with molecular modeling studies. Finally, the radical scavenging activity of carvacrol and thymol was evaluated by the ABTS radical scavenging assay. An improvement of this activity was observed upon encapsulation. Taken together, these results evidence that the encapsulation in CDs could be valuable for applications of carvacrol and thymol in food.

## Introduction

**Figure 1:**
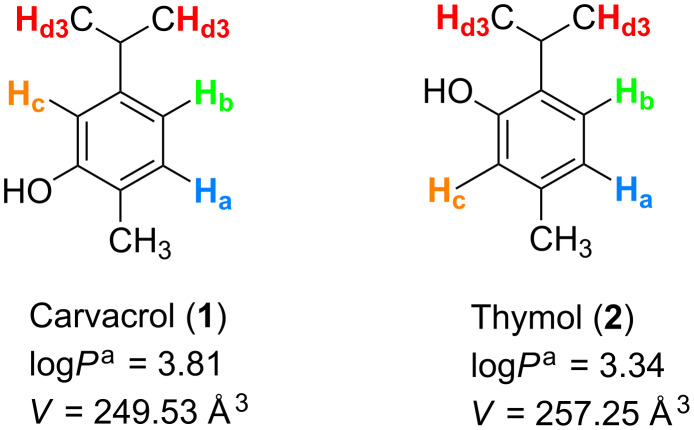
Chemical structures, log*P* values and molecular volumes (*V*) of carvacrol (**1**) and thymol (**2**). ^a^http://www.molinspiration.com/cgi-bin/properties. *V* = *M*/*d*NA, with M: molecular weight, d: density, NA: Avogadro’s number.

Carvacrol (2-methyl-5-(1-methylethyl)phenol, **1**) and thymol (5-methyl-2-(1-methylethyl)phenol, **2**) are monoterpenic phenol isomers (Figure 1) produced by several aromatic plants (oregano, thyme, savory, marjoram, etc.) [1]. They are generally recognized as safe (GRAS), approved by the US Food and Drug Administration for human consumption and included by the Council of Europe in the list of food flavorings [1–2].

These phenols are traditionally used at low concentrations as flavoring agents in food [3] and do not have any mutagenic or genotoxic effects [1]. They are cited by the European Commission among the essential oils components registered for use as flavoring in foodstuffs [2,4]. Recently, essential oils have received a growing attention as natural preservatives [5–6] especially in active packaging material for increasing the shelf-life of food products [7–8]. This is due to their potent activity against a broad range of natural spoilage bacteria, fungi and foodborne pathogens [9–10] as well as their pronounced antioxidant effect [11–12]. Consequently, they could be employed as alternatives to synthetic antioxidants such as butylated hydroxytoluene (BHT) or butylated hydroxyanisole (BHA), suspected to be carcinogenic [13–14]. However, the major drawbacks for their use in food are their low aqueous solubility that limits their homogenous dispersion and their contact with pathogens [15], their susceptibility for loss during storage or heat treatement [16] and their relatively high flavor impact and low flavor threshold that lead to the deterioration of food organoleptic quality [4]. Encapsulation in cyclodextrins (CDs) could overcome these limitations. Indeed, CDs have the ability to increase the solubility, protect encapsulated guests against a harmful environment, prevent interactions with food matrix components, generate controlled release systems, reduce off note development and maintain the true aromatic profile of the food [17–20]. CDs are crystalline, homogenous, non-hygroscopic cyclic oligosaccharides. The common native CDs contain 6, 7 and 8 D-(+)-glucopyranose units bound together by α(1→4) linkages and are referred to as α-, β- and γ-CDs [21]. The chair conformation of the glucose units results in a truncated shape of CDs with an external hydrophilic surface and a hydrophobic internal cavity that allows the encapsulation of hydrophobic guests by the formation of inclusion complexes. The substitution of hydroxy groups present on the rims of the torus leads to the production of CD derivatives with increased solubility and enhanced complexation ability [22–24].

Despite that several studies attempted to examine CD/**1** and CD/**2** inclusion complexes [25–33], little is known about the strength of interactions and the difference in the recognition ability of CDs for both isomers. Indeed, only the formation constant (*K*_f_) of the inclusion complex HP-β-CD/**2** (hydroxypropylated-β-CD/**2**) has been reported in literature [28].

Therefore, the present study aimed to determine the ability of CDs to encapsulate and solubilize **1** and **2**. The stoichiometry and *K*_f_ values of CD/**1** and CD/**2** inclusion complexes were determined using a competitive UV–visible method, phase solubility studies as well as ^1^H and DOSY ^1^H NMR titration experiments. An algorithmic treatment was applied to NMR results to calculate *K*_f_ values. This algorithm is the first attempt that associates numerous signals (chemical shifts and diffusions coefficients variations) from several entities of the guest molecule (different guest protons) simultaneously to calculate one *K*_f_ value. Then, 2D ROESY NMR was carried out to prove the encapsulation as well as to investigate the geometry of inclusion complexes. NMR studies were completed by molecular modeling investigations to illustrate the most energetically favorable conformation of inclusion complexes. Finally, the effect of encapsulation on the antioxidant properties of **1** and **2** was evaluated using the ABTS radical cation assay.

## Results and Discussion

### UV–visible competitive studies

Stoichiometries and *K*_f_ values of inclusion complexes of **1** and **2** with six CDs (α-CD, β-CD, γ-CD, hydroxypropylated-β-CD (HP-β-CD), randomly methylated β-CD (RAMEB) and a low methylated-β-CD (CRYSMEB)) were determined by an UV–visible competitive method using methyl orange (MO) as competitor [34]. Firstly, *K*_f_ values of CD/MO inclusion complexes were determined and were consistent with the literature [35]. Then, the competition method was applied. Variations in the absorbance spectra of MO were in good agreement with an 1:1 (CD:guest) stoichiometry proving that all studied CD/**1** and CD/**2** inclusion complexes present an 1:1 stoichiometry. This is coherent with generally observed results for aromatic monoterpenes [17–18,28]. *K*_f_ values (Table 1) were calculated based on the absorbance variations using an algorithmic treatment. Only a *K*_f_ value of the HP-β-CD/**2** inclusion complex, determined by fluorescence spectroscopy, was found in the literature (1400 M^−1^) [28]. The obtained *K*_f_ value is in good agreement with our results (Table 1).

**Table 1:** Formation constants *K*_f_ (M^−1^) of CD/carvacrol (**1**) and CD/thymol (**2**) inclusion complexes determined by the competitive UV–visible method at 25 °C.

*K*_f_ (M^−1^)	Carvacrol (**1**)	Thymol (**2**)

α-CD	454	107
β-CD	2620	1467
γ-CD	999	233
HP-β-CD	2154	1488
RAMEB	3564	3337
CRYSMEB	2421	2386

Compounds **1** and **2** differ only by the position of the hydroxy group on the aromatic cycle (Figure 1). Results showed that encapsulation of **1** and **2** occurred with all the six CDs. Nonetheless, both phenols were more readily recognized by β-CD and its derivatives as compared to α-CD and γ-CD. Our findings could be strengthened by the fact that the vigor of binding is highly influenced by the complementarity between guest and CD cavity. Molecules with aromatic ring structures would fit better within the β-CD cavity.

When comparing the performance of β-CD derivatives to the native CD, we observed a decline in the *K*_f_ value of HP-β-CD/**1** as compared to β-CD/**1** (the decrease in the *K*_f_ value of CRYSMEB/**1** was not significant <10%). This could be explained by the steric hindrance of the hydroxypropyl groups of HP-β-CD during the inclusion of **1** inside the cavity. RAMEB gave the most stable inclusion complexes with both **1** and **2**. This is due to that the methoxy groups of RAMEB are small and do not lead to a significant steric hindrance and that the methylation of β-CD hydroxy groups increases the hydrophobic character of the cavity which strengthen its binding to guests.

Concerning the influence of the position of the hydroxy group of **1** and **2** on their recognition by CDs, the former allowed the formation of relatively more stable inclusion complexes as demonstrated by higher *K*_f_ values. A tight steric complementarity between CD and guest is crucial to allow the formation of a stable inclusion complex. This is mainly controlled by the chemical structure of the encapsulated guest. *K*_f_ values generally increase for guests with an isopropyl moiety. Indeed, *p-*cymene [36] showed higher *K*_f_ values than toluene [37]. However, the comparison of *K*_f_ values for **1**, **2** and *p-*cymene with β-CDs showed that *K*_f_ values for **1** and *p-*cymene were similar while a decrease in the binding interactions for **2** was observed. Both **1** and **2** have a hydroxy group in addition to *p-*cymene. Thus, the decline in the *K*_f_ values observed for **2** could be attributed to the enhanced steric hindrance caused by the *ortho* position of the hydroxy group (Figure 1).

Moreover, **1** presents a relatively superior hydrophobic character than **2,** as expressed by log*P* values (Figure 1), which additionally reinforces hydrophobic interactions with the apolar CD cavity.

### Phase solubility studies

Phase solubility studies are widely used to evaluate the ability of CDs to increase the aqueous solubility of the guests. They also lead to the determination of diverse parameters involved in complex formation such as *K*_f_ value, complexation efficiency (CE), optimal molar ratio for solid inclusion complex prepration and increase in formulation bulk [38].

**Figure 2:**
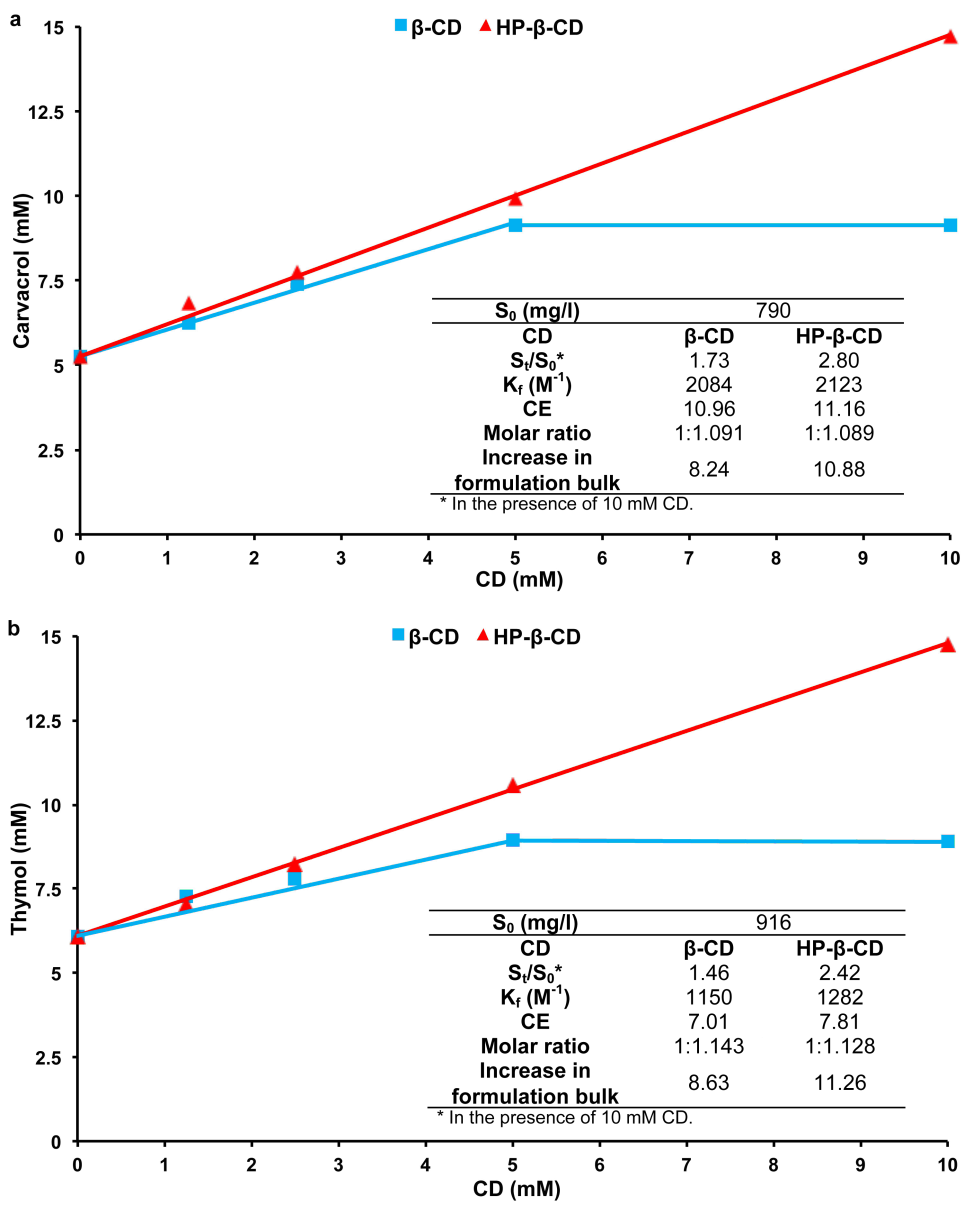
Phase solubility profiles of (a) CD/carvacrol (**1**) and (b) CD/thymol (**2**) inclusion complexes. Inset: Values of formation constants (*K*_f_), solubility enhancement ratio (*S*_t_/*S*_0_), complexation efficiency (CE), optimum molar ratio and increase in formulation bulk of each guest.

*K*_f_ values obtained by UV–visible competitive studies showed that β-CD and its derivatives form more stable inclusion complexes with **1** and **2** than α-CD and γ-CD. Moreover, HP-β-CD is the only β-CD derivative cited in the FDA’s list of Inactive Pharmaceutical Ingredients among the studied derivatives [39] and it previously showed a good solubilizing effect for natural aromas [18]. Consequently, phase solubility studies were performed only with β-CD and HP-β-CD. Results obtained with **1** and **2** at 25 °C are illustrated in Figure 2.

The aqueous solubility of **1** and **2** increased whith CD concentration. A_L_-type profiles were obtained with HP-β-CD while B-type profiles were observed in the case of β-CD with both monoterpenes. This could be attributed to the limited aqueous solubility of inclusion complexes obtained between β-CD and poorly soluble guests leading to their precipitation [38]. The slopes of A_L_-type and the linear segment of B-type profiles were less than one indicating the formation of 1:1 inclusion complexes in accordance with UV–visible results. *K*_f_ values were consequently calculated and were in good agreement with those obtained by the competitive UV–visible method. However, for both phenols, HP-β-CD did not give lower *K*_f_ values compared to β-CD as observed with the UV–visible competitive method. This could be explained by the better solubilizing potential of the β-CD derivative compared to the native one. Indeed, *K*_f_ values obtained from phase solubility profiles are generally apparent values that combine several effects on the guest solubility: inclusion complexation, self-association of poorly soluble guests, self-aggregation of CD:guest complexes, as well as non-inclusion interaction and micelles formation [38].

The solubilizing potential (*S*_t_/*S*_0_) of CDs was more important for **1** than **2** (Figure 2). This could be attributed to the lower intrinsic solubility of **1** as compared to its isomer. Results are in good agreement with the literature [18] where authors showed that solubilizing potential of CDs increased with the decrease in guest’s solubility.

For both phenols, CE and solubility enhancement were more important for HP-β-CD (Figure 2) confirming that β-CD derivatives are better solubilizers than native β-CD [18,38]. Optimal guest:CD ratios for solid inclusion complexes preparation as well as the increase in formulation bulks were subsequently calculated based on CE values and are presented in Figure 2. HP-β-CD led to a larger increase in the formulation bulk than the parent β-CD due to its greater molecular weight. Relatively high CE values were obtained. It has been reported that guests possessing log*P* between 1 and 4 frequently show good CE values in accordance with our findings [38]. High CE values and reasonable formulation bulk increase suggested that inclusion complexes of **1** and **2** could be potentially used in a solid dosage form for storage or further applications for both phenols [38].

### NMR spectroscopy

NMR spectroscopy has been widely employed to investigate CD inclusion complexes [40–41]. It is one of the most complete spectroscopic techniques because it allows a clear distinction between inclusion and other possible external interaction processes. Moreover, it gives direct information on the three-dimensional structure of inclusion complexes [42]. The protons of **1** and **2** are named according to Figure 1.

#### ^1^H and DOSY ^1^H NMR titration experiments

Generally, hydrogen atoms of CD and guest are affected by the inclusion resulting in a displacement of their chemical shifts (δ) and diffusion coefficients (D). ^1^H and DOSY ^1^H NMR spectra were recorded for free guests (**1** and **2**), pure β-CD (host) and for their inclusion complexes with guest/β-CD ratios ranging from 0.4 to 4. The concentration of guest was kept constant at 2 mM while the concentration of β-CD varied from 0.5 to 5 mM. The chemical shifts (δ) of β-CD protons in the free and complexed states are summarized in Table 2 at equimolar CD/guest ratios.

**Table 2:** ^1^H Chemical shifts (δ, ppm) corresponding to β-CD protons in the free and complexed states in the presence of equimolar amounts of either carvacrol (**1**) or thymol (**2)**.

β-CD ^1^H	Free	β-CD/carvacrol (**1**)		β-CD/thymol (**2**)	
	δ	δ	Δδ	δ	Δδ

H-1	5.11	5.08	−0.03	5.09	−0.02
H-2	3.70	3.67	−0.03	3.67	−0.03
H-3	4.00	3.93	−0.07	3.95	−0.05
H-4	3.62	3.61	−0.01	3.61	−0.01
H-5	3.89	3.76	−0.13	3.80	−0.09
H-6	3.91	3.83	−0.08	3.83	−0.08

A positive sign of Δδ ppm shows a downfield displacement and a negative sign an upfield displacement (Δδ = δ_complex_ − δ_free_).

The protons of the guest molecules were also affected by encapsulation. The chemical shifts (δ) and D of **1** and **2** protons at the different guest/CD ratios are tabulated in Table 3 and Table 4.

**Table 3:** Diffusion coefficients (*D*, 10^−10^ m^2^/s) and chemical shifts (δ, ppm) of carvacrol (**1**) protons in the presence of different β-CD concentrations.

β-CD (mM)	H_a_		H_b_		H_c_		H_d_	
	D	δ	D	δ	D	δ	D	δ
	
0	6.800	7.188	6.680	6.880	6.670	6.843	6.750	1.228
0.5	6.200	7.156	6.340	6.843	6.230	6.806	6.100	1.248
1.0	5.310	7.122	5.250	6.806	5.360	6.771	5.380	1.258
1.5	4.820	7.101	4.780	6.783	4.870	6.749	4.840	1.268
2.0	4.570	7.084	4.540	6.766	4.540	6.733	4.530	1.276
2.5	4.210	7.068	4.180	6.749	4.150	6.716	4.150	1.285
5.0	3.440	7.035	3.460	6.713	3.380	6.681	3.360	1.302

**Table 4:** Diffusion coefficients (*D*, 10^−10^ m^2^/s) and chemical shifts (δ, ppm) of thymol (**2**) protons in the presence of different β-CD concentrations.

β-CD (mM)	H_b_		H_a_		H_c_		H_d_	
	D	δ	D	δ	D	δ	D	δ
	
0	6.880	7.261	6.870	6.854	6.830	6.791	6.760	1.203
0.5	6.080	7.219	6.120	6.806	6.110	6.780	6.070	1.212
1.0	5.630	7.185	5.710	6.785	5.570	6.770	5.550	1.222
1.5	5.180	7.152	5.320	6.734	5.100	6.764	5.010	1.231
2.0	4.610	7.132	4.760	6.711	4.740	6.759	4.780	1.235
2.5	4.240	7.108	4.210	6.684	4.170	6.752	4.370	1.239
5.0	3.580	7.063	3.650	6.632	3.620	6.742	3.630	1.251

We note that no new peak appeared in the inclusion complexes spectra. This indicated that the inclusion of **1** and **2** in CD is a fast exchange process that takes place on the NMR timescale.

In the presence of either **1** or **2**, the protons of β-CD underwent changes in their chemical shifts (δ) (Table 2). The upfield shifts of H-1, H-2 and H-4 protons of β-CD were marginal as compared to those observed for H-3, H-5 and H-6. This indicated that both guests only interact with the inner cavity of CD. Moreover, the clear upfield shift of the H-6 proton of CD could be explained by the deep insertion of guests and showed that interactions occurred between **1** and **2** protons and the narrow side of CD due to steric hindrance. Particularly, the shift of H-3, H-5 and H-6 to higher magnetic fields could be attributed to magnetic anisotropy effects due to their location near to the aromatic ring of the guests which is rich in π-electrons [43].

The protons of **1** and **2** were also affected by the presence of β-CD (Table 3 and Table 4). A progressive upfield shift of the aromatic protons (H_a_, H_b_ and H_c_) of **1** and **2** was observed when increasing the CD concentration. Other protons of the guest showed progressive downfield shifts with less pronounced magnitude than those observed for the aromatic protons.

This upfield shift indicated that the aromatic protons of **1** and **2** are mainly involved in the hydrophobic interactions with the interior of the CD cavity [44–45]. Additionally, this revealed some conformational changes generated by the inclusion of **1** and **2** in the CD. The downfield shift observed for other guests’ protons is due to a variation in the polarity of their micro-environment when **1** and **2** are inside the CD cavity [46]. This also indicated a shielding effect due to the interactions between guest and CD [47], particularly by van der Waals interactions [48].

These observations suggested that the whole guest molecule is involved in the binding process to CD with the aromatic cycle of both **1** and **2** playing the prominent role in the inclusion process and being embedded in the center of the CD cavity near to oxygen atoms.

**Figure 3:**
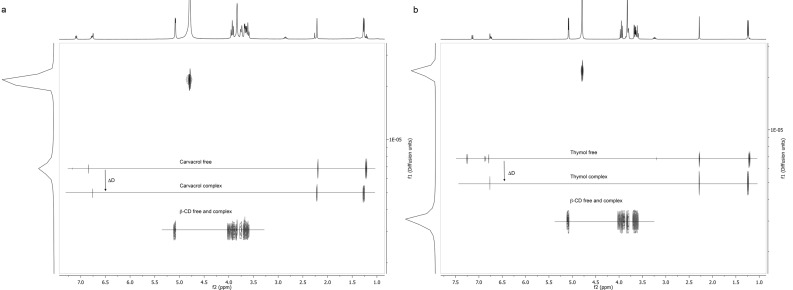
2D DOSY NMR spectra of (a) β-CD, carvacrol (**1**) and β-CD/carvacrol (**1**) inclusion complex and (b) β-CD, thymol (**2**) and β-CD/thymol (**2**) inclusion complex.

DOSY experiments also reveal the intermolecular interactions in solution by observing the variation in the intrinsic diffusion coefficients (*D*) of compounds upon interactions. The *D* values of **1** and **2** protons at the different guest/CD ratios are given in Table 3 and Table 4. The results for the 2 mM solutions of β-CD, guests (**1** and **2**) and the corresponding inclusion complexes are graphically depicted in the 2D DOSY plot in Figure 3. In these spectra, the f1 dimension shows the diffusion coefficient expressed as log*D* and the f2 stands for the chemical shift (δ). f1 is specific for each molecule thus moieties that belong to the same entity will appear in the same f1 row.

CD and guests possess their own *D* values in the free state. *D* is directly related to the molecular weight and size of each molecule. The guests molecules presented higher *D* values than CD in agreement with the fact that the guests are smaller than CD [49]. During the DOSY experiments for β-CD/**1** and β-CD/**2** inclusion complexes, *D* values of β-CD were relatively unaffected by the presence of neither **1** nor **2**. This is due to the small relative mass changes between the free and the complexed macrocycle. Meanwhile, the *D* values of both encapsulated **1** and **2** decreased (Table 3, Table 4 and Figure 3). This proved that **1** and **2** are included in the CD cavity and diffuse slowly.

**Figure 4:**
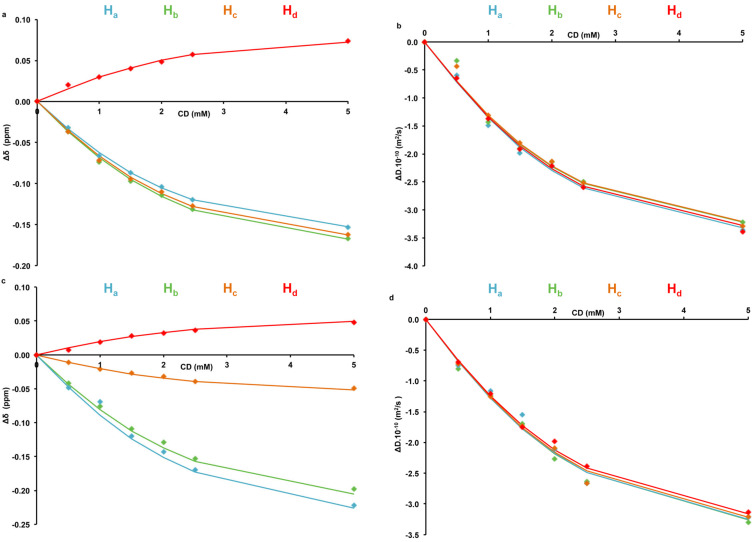
Representation of chemical shifts variations (Δδ) of a) carvacrol (**1)** and c) thymol (**2)** protons and diffusion coefficients variations (ΔD) of b) carvacrol (**1)** and d) thymol (**2)** protons with various β-CD concentrations. Experimental results are represented as filled diamonds and theoretical data are illustrated as solid lines.

Finally, variation of chemical shifts (Δδ) and diffusion coefficients (Δ*D*) were plotted as a function of CD concentration for both guests (Figure 4). A global analysis was applied to determine the host/guest affinity. A unique *K*_f_, together with the maximum shifts of each signal, were used to fit simultaneously theoretical and experimental data for all considered Δδ and Δ*D*. The obtained *K*_f_ values for β-CD/**1** and β-CD/**2** were equal to 1736 M^−1^ and 1344 M^−1^, respectively. These values are consistent with the UV–visible competitive method and phase solubility studies.

#### 2D ROESY NMR

2D ROESY spectroscopy is a very useful technique for describing the real structure of CD inclusion complexes and indicating the exact positioning of guest inside the CD cavity. It is based on the observation of the nuclear Overhauser effect (NOE) between the protons of the guest and that of the CD that take action in the inclusion process [42]. The presence of NOE cross correlation peaks between the protons of guest and CD indicates space couplings and confirms that protons are close in space (<4Å).

**Figure 5:**
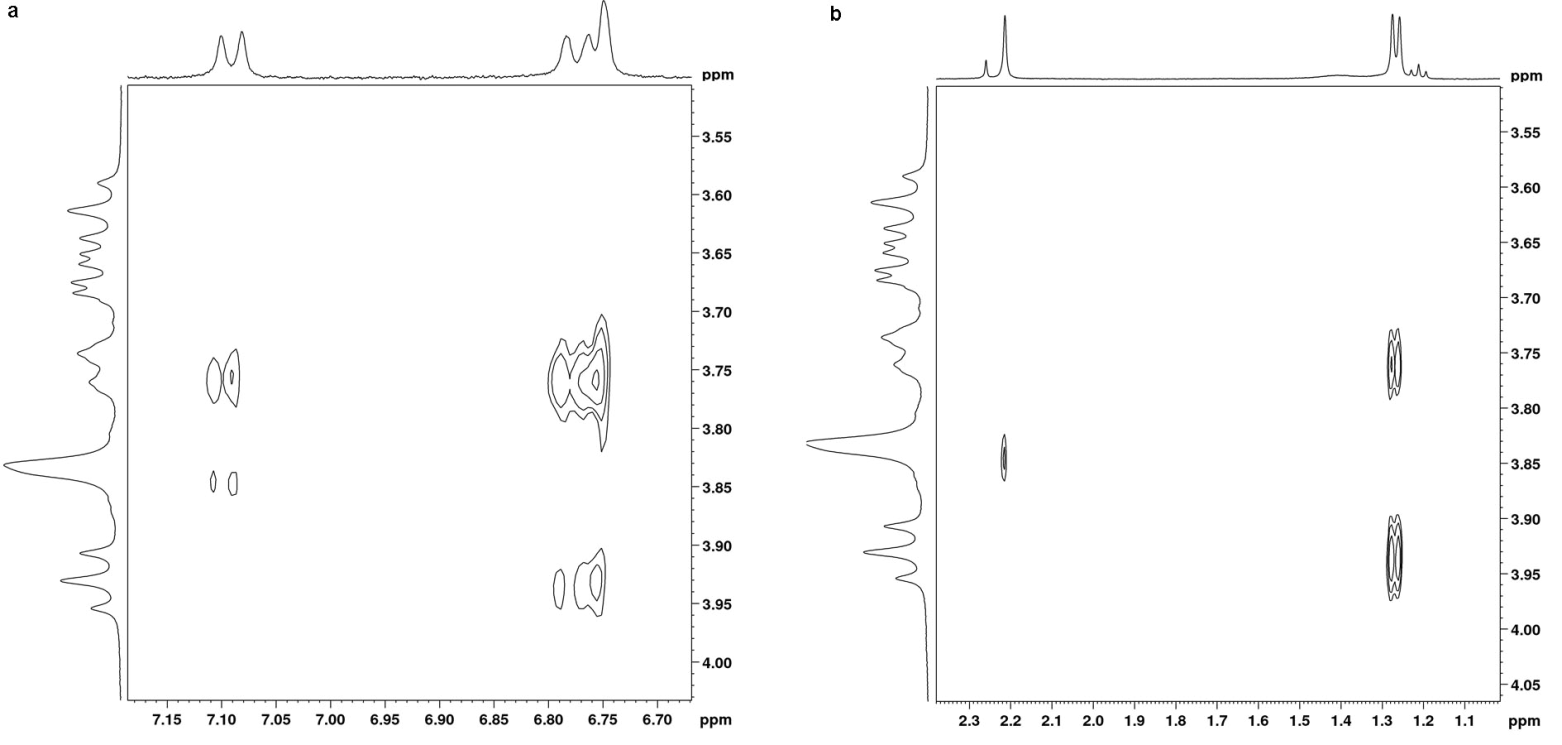
2D ROESY plots of β-CD/carvacrol (**1**) complex in D_2_O showing the NOEs between the H-3 and H-5 protons of β-CD and (a) the aromatic protons and (b) the aliphatic protons of carvacrol (**1**).

**Figure 6:**
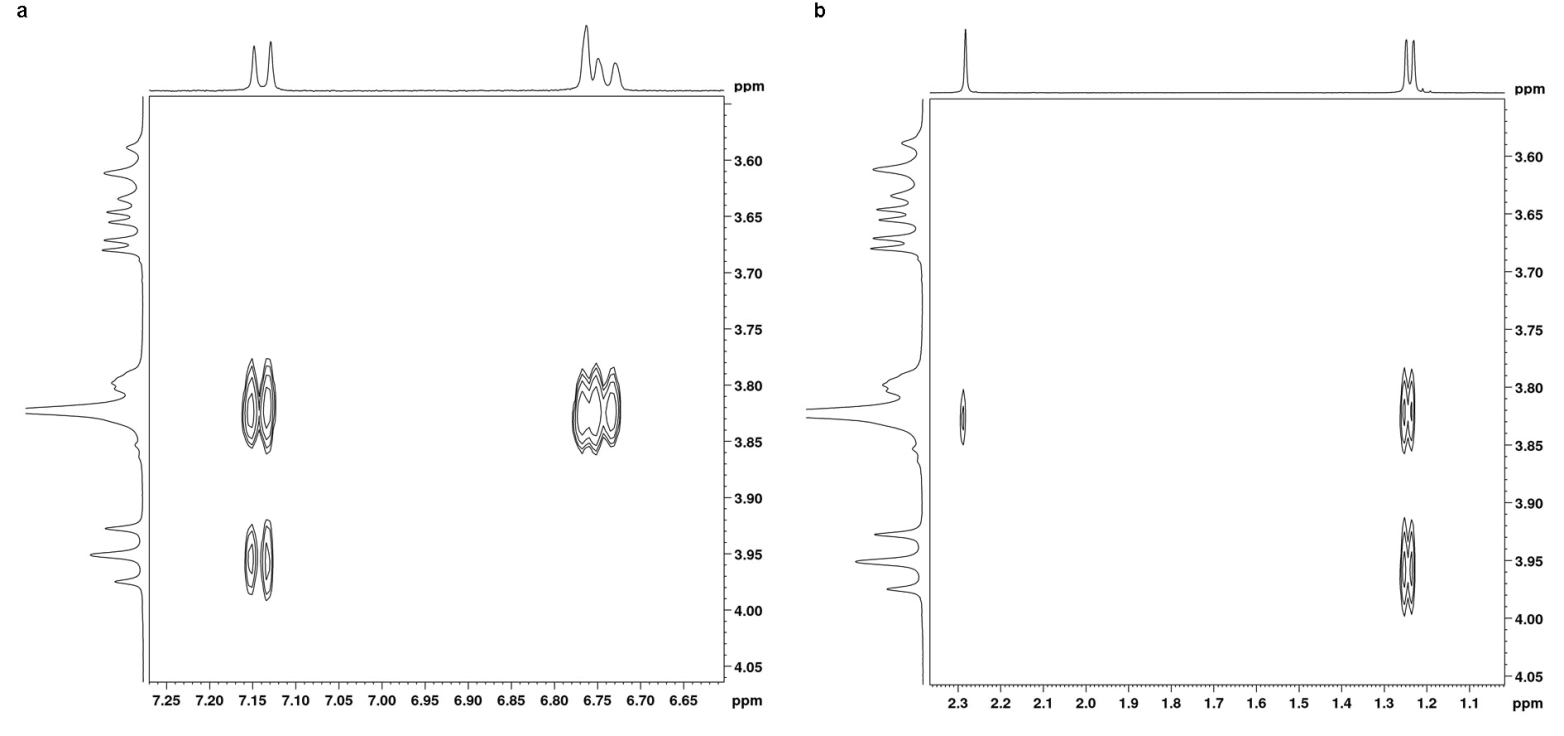
2D ROESY plots of β-CD/thymol (**2**) complex in D_2_O showing the NOEs between the H-3 and H-5 protons of β-CD and (a) the aromatic protons and (b) the aliphatic protons of thymol (**2**).

We performed 2D ROESY experiments for inclusion complexes of both β-CD/**1** and β-CD/**2** prepared at equimolar ratios. Partial contour plots of the ROESY spectra of inclusion complexes are shown in Figure 5 and Figure 6 for **1** and **2**, respectively.

First, the absence of any NOE cross correlation peaks between **1** and **2** protons and H-1, H-2 and H-4 protons of β-CD ruled out any significant interaction between guests and the external surface of β-CD at equilibrium in agreement with ^1^H NMR results.

For both guests, ROESY spectra showed two important sets of intermolecular cross-peaks. The first was observed between β-CD cavity protons (H-3 and H-5) and aromatic protons of **1** and **2** (H_a_, H_b_ and H_c_) and was stronger than the second one between the protons of the β-CD cavity and those of the methyl and isopropyl groups of **1** and **2**. This confirmed that, for both guests, the aromatic ring was deeply included in the β-CD cavity and that encapsulation occurred mainly through interactions with their phenyl moiety. But, it also pointed out that other guests’ protons are involved in the complexation.

We can particularly see that H_b_ and H_c_ protons of **1** showed NOE cross peaks with both cavity protons while the H-a proton of **1** displayed NOE correlation peaks only with the H-5 proton of the CD cavity. Also, NOE cross peaks were observed between the H_a_ and H_c_ protons of **2** and both CD cavity protons but the H_b_ proton of **2** exhibited NOE correlations only with the H-5 proton of the CD cavity. This indicated that the H_a_ proton of **1** and H_b_ proton of **2** are oriented toward the narrower primary rim of β-CD.

Moreover, non-aromatic (aliphatic) protons of both **1** and **2** also revealed cross correlation peaks with CD protons. NOE cross peaks were observed between the protons of the isopropyl group of the guests and both protons H-3 and H-5. This indicated a partial penetration of the isopropyl group into the CD cavity. Moreover, the H-6 proton of β-CD showed cross peaks only with the protons of the methyl group of both **1** and **2** but not with those of the isopropyl moiety. This showed that, for both guests, the methyl group is pointed towards the primary narrower rim of the CD host.

According to these observations, it became possible to estimate the orientation of **1** and **2** inside the β-CD cavity: the methyl group of both guests is oriented toward the narrower rim of the CD cavity whereas the isopropyl moiety points to the wider rim.

### Molecular modeling

**Figure 7:**
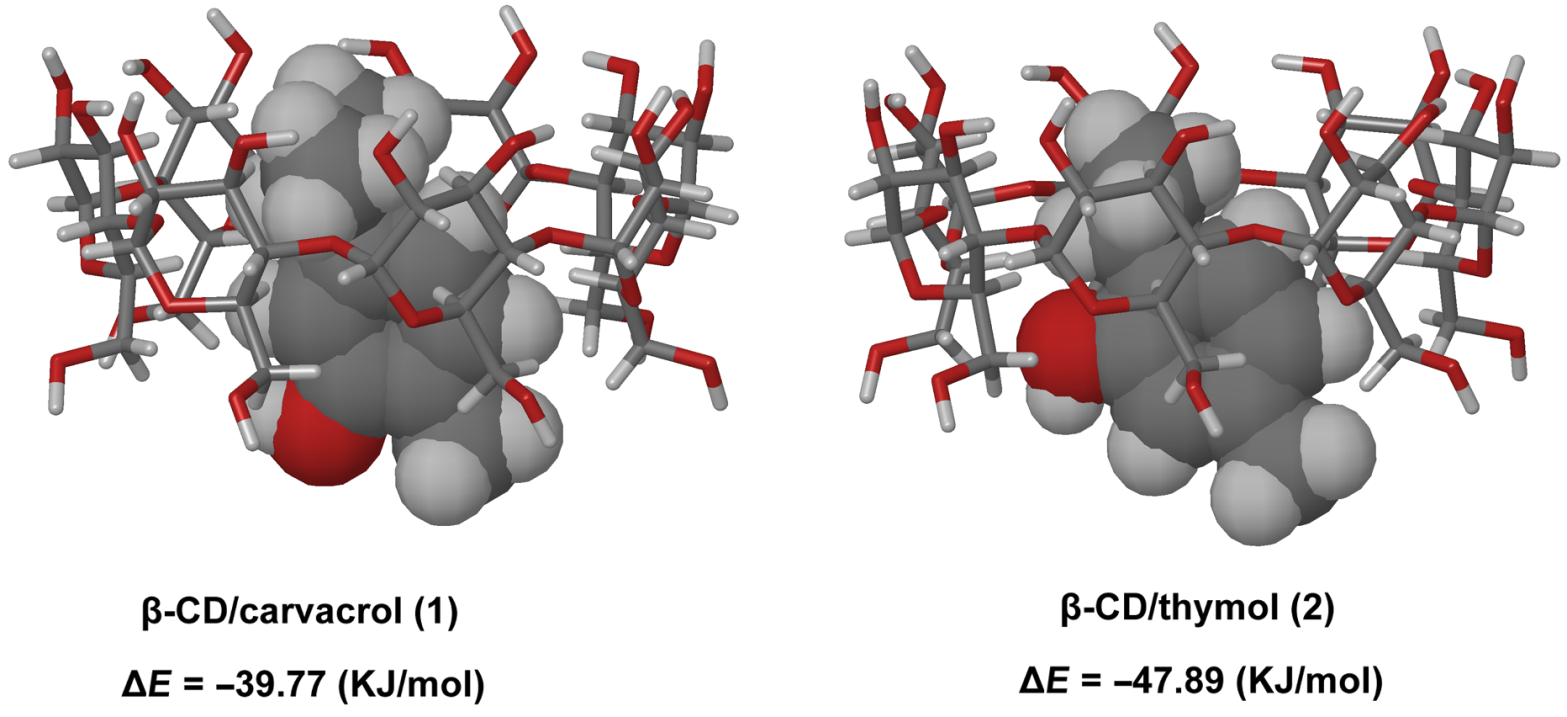
Representation of the most stable CD/guest inclusion complex conformers.

A molecular modeling study was performed to rationalize the NMR results, find out the most probable conformations of the inclusion complexes in solution and illustrate their 3D structures. The most stable inclusion complexes conformers, presenting the weakest relative binding energies (Δ*E*) values, are illustrated in Figure 7.

Firstly, results showed that **1** and **2** could form inclusion complexes with β-CD with the aromatic cycle embedded inside the lipophilic cavity. This supported the fact that hydrophobic forces play a leading role in inclusion complex formation. Although Δ*E* values clearly illustrate the stability of each inclusion complex, it has to be underlined that such theoretical energies cannot be directly compared to *K*_f_ values, as the entropic part of the inclusion phenomena is not simulated.

In addition, the applied conformational research method showed that different conformers probably co-exist, since various structures with Δ*E* values quite close to those of the most stable conformers were obtained for both guests. On a structural point of view, these results are also consistent with the experimental NMR data showing that the most stable conformers for β-CD/**1** and β-CD/**2** resulted from a preferential inclusion mode; guests penetrate the CD via their methyl moiety and the isopropyl group of both is pointed to the secondary wider rim.

It is interesting to note that the H_c_ proton of **2** protruds outside the cavity (Figure 7). This might result from the fact that the hydroxy group of **2** comes close to the primary hydroxy groups of CD to form hydrogen bonds that further stabilize the inclusion complex. This leads to the projection of the H_c_ proton of **2** outside the cavity. This observation could explain why, during the ^1^H NMR titration experiments, the H_c_ proton of **2** showed less pronounced chemical shift variations (Δδ) (Figure 4) upon encapsulation as compared to H_a_ and H_b_ protons.

### Radical scavenging activity

Both compounds **1** and **2** are described as potent free-radical scavengers [29]. Moreover, it is well accepted that a wide variety of essential oils possess important antioxidant activities due to their high content in **1** and **2** [50]. In this work, the effect of encapsulation on the antioxidant activity of **1** and **2** was evaluated. The ABTS^•+^ assay is commonly applied to determine the antioxidant activity of CD inclusion complexes [51–54]. We performed this test to determine the radical scavenging ability of **1** and **2** as well as the activity of their corresponding β-CD and HP-β-CD inclusion complexes. Trolox was used as reference and the results were expressed as Trolox equivalent antioxidant capacity TEAC (μmol Trolox/g of guest).

**Figure 8:**
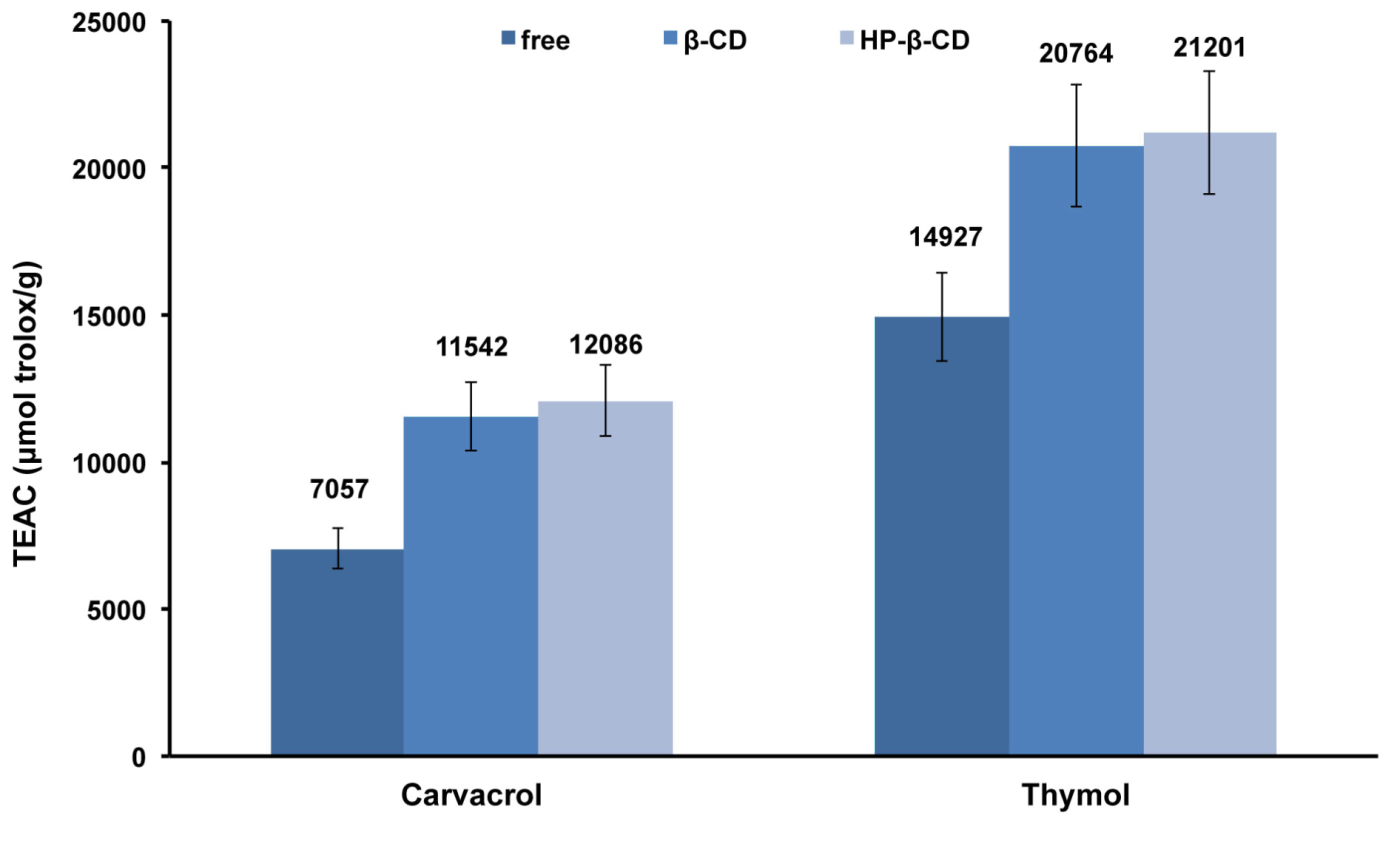
Effects of β-CD and HP-β-CD on the TEAC (μmol Trolox/ g of guest) of carvacrol (**1**) and thymol (**2**) by using ABTS^•+^ assay.

As can be seen in Figure 8, both phenols exhibited anti-ABTS^•+^ scavenging activity with **2** being more potent. This could be attributed to the difference in the position of aromatic cycle substituents, which affects the stability of the resulted phenoxyl radical upon reaction of guest with ABTS^•+^. Similar TEAC value for **1** was reported in the literature [32].

We should note that a decrease of ABTS^•+^ absorbance was observed when the assay was carried out with CDs alone. This fact could be attributed to the inclusion of ABTS^•+^ inside the CD cavity in agreement with literature [51]. We then compared the activity of **1** and **2** to their corresponding inclusion complexes. As we can see in Figure 8, inclusion complexes showed higher radical scavenging activities than free molecules. The increased antioxidant activity could be attributed to the encapsulation of **1** and **2** in CDs [51]. Inclusion in CD cavity could protect and stabilize the formed phenoxyl radicals after reaction with ABTS^•+^ leading to an enhanced activity by delaying its oxidation. It has been also demonstrated that CDs could act as secondary antioxidants and improve the activity of antioxidants [55]. Altogether data indicated that CDs could increase the half-life of antioxidant compounds and broaden their applications.

## Conclusion

In this work, we clearly demonstrated that CDs could successfully encapsulate **1** and **2**. Experimental and theoretical results showed that all inclusion complexes have a 1:1 CD:guest stoichiometry and that the molecular structure of the guest affected its binding ability to CD. *K*_f_ values determined by an UV–visible competitive method, phase solubilty studies and ^1^H and DOSY ^1^H NMR titration experiments were consistent. 2D NMR and molecular modeling studies revealed the geometry of the most stable inclusion complexes. Encapsulation of **1** and **2** in CDs made them more soluble in aqueous systems than their free forms and improved their radical scavenging activity. Thus, CD/**1** and CD/**2** inclusion complexes could be used in food formulations as flavoring and antioxidant agents.

## Experimental

### Materials

Carvacrol (**1**), thymol (**2**), Trolox and K_2_S_2_O_8_ were purchased from Aldrich. Methyl orange (MO) was purchased from Acros Organics. CRYSMEB (DS = 4.9) was provided from Roquette Frères (Lestrem, France), α-CD, β-CD, γ-CD, HP-β-CD (DS = 5.6) and RAMEB (DS = 12.6) were purchased from Wacker-Chemie (Lyon, France). All products were of analytical grade and were used as received. Distilled deionized water was used all over the study.

### UV–visible competitive studies

Formation constants (*K*_f_) values of inclusion complexes were determined by an UV–visible competitive method (or spectral displacement method) using the azo dye competitor MO [34]. This method requires a previous determination of *K*_f_ values of CD/MO inclusion complexes by a direct titration method. The competitive method was applied by adding **1** and **2** to a solution containing known concentrations of CD and MO. This addition induced an absorbance increment leading to the assessment of *K*_f_ values for the CD/**1** or CD/**2** inclusion complexes. The MO concentration was fixed to 0.1 mM and spectra were recorded between 520–530 nm with a 1 cm thick quartz cuvette using an UV–visible dual-beam spectrophotometer (Perkin Elmer Lambda 2S) at 25 °C. MO shows optimal differences in absorbance in this wavelength range between its free and complexed forms. Aiming to avoid any spectral influence of diffraction phenomena, the *K*_f_ values were calculated using an algorithmic treatment applied to the first derivatives of UV spectra. Experiments were done in triplicate.

### Phase solubility studies

Phase solubility studies were carried out as described by Higuchi and Connors [56]. Excess amounts of **1** or **2** were added to 1 mL of CD solution at different concentrations ranging from 0 to 10 mM. The obtained mixtures were shaken at 25 °C for 24 h then filtered through a 0.45 μm cellulose filter. The concentrations of **1** or **2** in the filtrate were determined spectrophotometrically at 275 and 277 nm, respectively. Phase solubility profiles were obtained by plotting the solubility of **1** or **2** as a function of CD concentration. The *K*_f_ value of each inclusion complex was calculated from the linear segment of the corresponding phase solubility profile using the following equation:

[1]
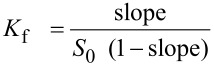


where *S*_0_ is the intrinsic solubility of **1** or **2** when no CD was added and the slope is the slope of the phase solubility profile. The solubilizing capacity of CD was estimated by the complexation efficiency (CE) parameter. CE was calculated from the slope of the phase solubility profile and is equal to the complex to the free CD concentrations ratio:

[2]
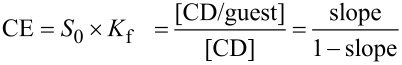


where [CD/guest] is the concentration of the dissolved inclusion complex and [CD] is the concentration of free CD. Consequently, the CE allowed the evaluation of guest:CD optimal preparation ratio as follows:

[3]
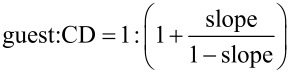


The correlation between CE and the molecular weights of CD or guest leads to the evaluation of the increase in formulation bulk that can be calculated as follows:

[4]
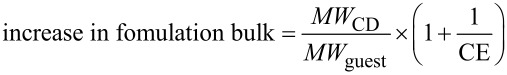


where *MW*_CD_ and *MW*_guest_ are the molecular weights of CD and guest, respectively. All preparations and experiments were done in triplicate.

### NMR experiments

All NMR experiments were carried out in D_2_O (4.79 ppm) and were recorded on a Bruker Avance III spectrometer at 400 MHz (9.4 T), equipped with a multinuclear *z*-gradient BBFO probe head capable of producing magnetic field pulse gradients in the *z*-direction of 48.15 G·cm^−1^. Throughout all experiments, the probe temperature was maintained at 300 K and standard 5 mm NMR tubes were used. The ^1^H spectra were recorded by averaging 32 scans, with a digital resolution of 0.30 Hz. ^1^H NMR spectra were recorded for six samples containing mixtures of β-CD and guests with guest/β-CD molar ratios ranging from 0.4 to 4.

2D NMR experiments were carried out for inclusion complexes prepared by mixing β-CD and guest in a 1:1 molar ratio at a concentration of 2 mM.

2D ROESY spectra were acquired with a mixing time of 600 ms during spin-lock with 64 scans using the States-TPPI method with a 1024 K time domain in F2 and 256 experiments in F1.

2D DOSY spectra were performed using the bipolar longitudinal eddy current delay (BPPLED – bipolar pulsed field gradient longitudinal eddy delay) pulse sequence. The pulse gradients were incremented in 16 steps from 2 to 98% of the maximum gradient strength in a linear ramp. Diffusion times and gradient pulse durations were optimized for each experiment in order to achieve a 95% decrease in resonance intensity at the largest gradient amplitude: Typically, diffusion time between 75 and 300 ms, gradient strength between 0.55 and 3 ms, spoil gradient strength of 0.6 ms, and longitudinal eddy current of 5 ms. After Fourier transformation, phase and baseline correction, the diffusion dimension of the 2D DOSY spectra was processed by means of the Bruker Dynamics Center software (version 2.1.9). The diffusion constants were calculated by exponential fitting of the data belonging to individual columns of the 2D matrix. The software gave the mean value of the diffusion coefficient.

The *K*_f_ values were obtained by global analyses of ^1^H and DOSY ^1^H data, using a non-linear treatment. Briefly, for each prepared solution, the inclusion complex concentration may be expressed as follows for a 1:1 stoichiometry:

[5]
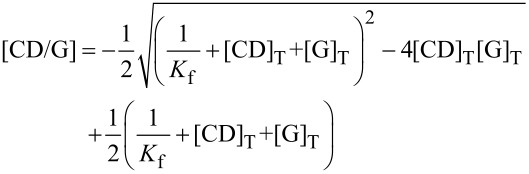


With [CD/G], [CD] and [G] being the complex, CD and Guest concentrations, respectively. The subscript _T_ stands for total.

Then, the variation of chemical shifts (Δδ) and diffusion coefficients (Δ*D*) were calculated according to:

[6]
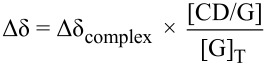


[7]
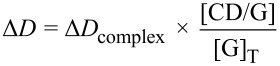


With Δδ_complex_ and Δ*D*_complex_ being respectively the chemical shift variation and the diffusion coefficient variation, between the free and complexed forms of the guest.

The squared differences between theoretical and experimental data are then summed over all solutions and over all guests’ ^1^H and DOSY signals. These Δδ and Δ*D* differences are weighted relatively to each other in order that chemical shift (δ) and *D* generate equal sum of squared differences, in such a way that both signals contribute significantly to the determination of *K*_f_. A Newton–Raphson procedure finally minimizes the sum of the squared differences by varying the unique *K*_f_ value and each Δδ_complex_ and Δ*D*_complex_.

### Molecular modeling

The determination of possible inclusion complex conformations was carried out by a conformational Monte Carlo research method using the MMFFs force field in the presence of water (GB/SA implicit model) with the generation of 5000 conformations (FMNR conjugate gradient minimization convergence fixed to 0.01 kJ Å^−1^ mol^−1^). Prior to docking and simulations, the structures of **1** or **2** were constructed manually and minimized. The host β-CD structure was a non-distorted symmetrical shell that was maintained rigid during the conformational search. Guests **1** or **2** were allowed to freely rotate and translate during the search. The total energy difference (Δ*E*, kJ/mol) between inclusion complexes and the sum of their individual components (CD and **1** or CD and **2**) in their optimized fundamental states was calculated for the most stable conformers. Δ*E* was used as the theoretical parameter to evaluate the complexation energy of the inclusion complex.

### ABTS radical scavenging method

The ABTS (2,2'-azino-bis(3-ethylbenzothiazoline-6-sulfonic acid) radical cation (ABTS^•+^) scavenging method was used to determine the radical scavenging potency of free and encapsulated **1** and **2**. This method relies on the capacity of an antioxidant to scavenge and reduce ABTS^•+^ into its colorless reduced state. The ABTS^•+^ was generated by reacting the ABTS salt (7 mM) with K_2_S_2_O_8_ (2.45 mM) in water at room temperature in the dark for 12–16 h. A diluted ABTS^•+^ solution was then prepared in water to obtain an initial absorbance of 0.75 ± 0.2 at 730 nm using an UV–visible dual-beam spectrophotometer (Perkin Elmer Lambda 2S) with a 1 cm thick quartz cuvette. Aliquots of free and encapsulated **1** or **2** were added to 2 mL of ABTS^•+^ containing solutions. The solutions were shaken in the dark for 1 hour at 25 ± 0.1 °C. The absorbance was measured at 730 nm. Blank samples contained ABTS^•+^ alone or in the presence of 10 mM of CD. The radical scavenging activity was expressed as Trolox equivalents TEAC (μmol Trolox/g of G) by using a Trolox calibration curve prepared for a concentration range of 2.5–25 μM. All analyses were done in triplicate.
